# The Theory of Reasoning
*The Theory of Reasoning. By Samuel Bailey. Longman & Co. 1851.


**Published:** 1851-10-01

**Authors:** 


### Art. II.—THE THEORY OF REASONING.*

Like every other branch of learning, Logic has not escaped the general scrutiny which has been applied, during the present century, and especially of late years, to the most established systems of knowledge, and to the master-pieces left us by the greatest names. Aristotle has reigned over more countries than ever his renowned pupil, Alexander, over-ran by the prowess of his arms; and while the conquests of the latter have left scarcely any traces on the map of the world, the influence of the former was absolute, for many ages, over the most enlightened nations of the earth; and it has by no means ceased, even now, to be partially recognised. His logical treatises have been the admiration of successive generations: and though the "Organon," under which title they have been comprised, contains much which the greatest advocates of their author acknowledge to be extra-logical, the main principle on which the system is founded may be said to have maintained itself, in one form or another, down to the present moment in which we write. Dugald Stewart only did justice to Aristotle when he said that "the conception and execution of so vast a plan as that in which the philosopher has included all reasoning, are such, that Aristotle's logical writings will ever form a proud and imperishable trophy to his genius." Many have been the attempts to overthrow the throne of the Stagyrite in his domain, but we may safely say that no one has yet succeeded in fairly wresting the sceptre from his hand. Is not this because, whatever be the faults which Aristotle is guilty of in detail, the main principles on which his claim to the empire of logic rests are inviolable? Whatever answer may be given to this question, we are quite willing to lend a candid ear to any kind of speculations on the subject, prepared as we are to anticipate that they will turn out to be really proposals of emendation in detail, rather than what we must be compelled to admit as fundamentally and essentially distinct principles.

The neglect of logical studies in this country, till a recent period, was very much owing, we think, to the influence of a few great names, among whom may be placed that of Locke. His prejudice against logic was hardly dealt with too severely by Leibnitz, when he said of a philosopher from whose psychological system, in general, he no doubt greatly differed, though he treated him in criticizing it with exemplary candour—*sprevit Logicam, non intellexit.* Murray's Logic at Dublin, Duncan's in Scotland and at Cambridge, and Aldrich's *Compendium* at

* The Theory of Reasoning. By Samuel Bailey. Longman & Co. 1851.

Oxford, supplanting much better and more learned works, all testify the low ebb to which logical studies had sunk at the outset of the present century, in the main seats of learning in the three kingdoms. There is no question that the credit of having revived these studies is mainly due to Dr. Whately, the present Archbishop of Dublin, whose "Elements of Logic" decidedly form an epoch in the history of this science, though his work is by no means on a level, for learning and fundamental research into the laws of thought, with the German, or even the scholastic writers on the subject. It is a popular work, well-calculated to draw general attention to logic; for an easier book on science no boy in his teens need ever wish to open: but neither historically nor scientifically, is it so satisfactory as to promise anything like a very lengthened reign. The truly masterly, surpassingly learned, and wonderfully acute and elaborate criticism (with which the public are now familiar) of Whately's book, from the hand of Sir W. Hamilton, may almost make the reader tremble for any one who enters anew, as an author, on the high field of logic. Here, however, Mr. Bailey has ventured; and the attention which he has already paid to *metaphysical* subjects, induces us to follow him into this arduous arena with somewhat less apprehension than we should have felt for any man who should now come forward with a new theory of reasoning. For really we are having so many "logics," containing so many different views, that it is not very easy to suppose any fundamentally new principle, whatever modifications of old ones may be possible. Mr. Bailey, however, is not a mere novice. He is already a psychologist. No man ought to write on logic who has not studied a good deal in this direction, generally. We say "metaphysical subjects," because we hold logic to be fundamentally metaphysical. The Germans have done right in regarding psychology as divisible into "experimental" and "metaphysical." Of the former we have an example in the laws of association, which we only know in their details, by experience. Of the latter we have instances in all those forms of thought which are primary, and incapable of analysis—forms of thought by which we cannot help being ruled, though we cannot prove their validity either by demonstration or by experience. In fact, they are neither deductive nor inductive. Thus, for instance, that every change in the universe around us must have its cause, is a principle felt to be as certain, or thereabouts, as our own individual existence; but no man can prove it, in any way. We believe it, even from early childhood, because we cannot help believing it, and we stake everything on it. It is a "metaphysical" truth; and on such truths does logic itself rest. But we must now address ourselves to Mr. Bailey. We are glad to perceive, at all events, that he does not set out with the extraordinary and unheard of heresy of Professor Blakey—that of cashiering all demonstrative reasoning from the domain of logic, (of which it is the most perfect example,) and limiting the dialectic art and science wholly to moral subjects!

Mr. Bailey, in this work, proposes to himself to give a connected and consistent view of the theory of reasoning, and of the relation in which the several parts of it stand to each other. He states that his views differ as a whole, and in some of their details, from any theory hitherto promulgated; but he adds, very properly, that "there can be no merit in any difference from former works, unless that difference is founded in truth." He informs us that he "designed at first to make the treatise wholly expository; but the number of unsettled questions on which he had to touch, forced him more extensively into criticism and controversy than he had originally contemplated entering. In such a work it was especially impossible not to advert to the scholastic logic; and as his theory is at variance with some of its fundamental principles, he has had occasion to comment upon it at considerable length."

We have no desire to enter very critically into those preliminary or adjunct remarks which are rather incidental to the work than essential parts of the author's views of argument or syllogism. Nevertheless, accuracy of statement, and consistency of nomenclature, are of the utmost moment in all subjects connected with psychology, including, of course, those important branches of it, logic and ethics. We are not aware of any standard author on mental philosophy who would identify "recollection" and "mere conception," as Mr. Bailey evidently does in his first chapter. A man may form a *conception* of an object never before known to him even in thought; while recollection is evidently a distinct case of remembrance, attended with volition, and involves a similar previous state of consciousness. But let this pass. Our author defines reasoning to be the "determination of the mind to the belief of something beyond its actual perception or knowledge;" or, at all events, he says, "this determination is obviously what is termed reasoning." We should offer some remarks on this passage, if we were not desirous of suspending our criticism in order to allow the author to speak more fully for himself. He goes on to say, "There is, however, another mental operation to be noted, which consists, not in our being led to believe, or in our inferring from what we perceive and know, something else neither perceived nor known; but in our being led to discern some fact, not directly manifest, through the medium of some other fact or facts in which it is implied." In illustration of the above, the fifteenth proposition of Euclid's first book is referred to, proving the equality of the vertical angles, where two straight lines intersect, by the construction necessarily involving a common supplement to these angles. "Here we do not infer the existence or the happening of something past, or future, or absent; but we are led to discern something not directly obvious, by an arrangement of propositions expressive of facts, each of which implies its successor." Waiving all discussion as to the propriety of the terms which Mr. Bailey employs in designating these two modes of reasoning, and especially the use of the term *fact* in regard to demonstration, in which the construction is merely an example of an infinite number of cases, which are all concluded *à priori*, it is evident that he here points out the old distinction between that kind of reasoning which is only probable, however it may approach to moral certainty, and that which is strictly necessary as being based on demonstration. Our author, however, prefers designating the former kind of reasoning by the appellation *contingent*.

Next follows a more particular inquiry into the nature of "contingent" reasoning. "We have observed that the tide, in ebbing, has left the sea-weed high on the beach. I recollect this fact; and on seeing the sea-weed left high on the sea-beach, I now conclude that the tide has washed it there." In the same manner we infer that the "gay people walking on the beach will, sooner or later, die. In the first example, a past event is inferred from other past facts; in the second, future events are inferred from past events." Our author justly adds, that it is the resemblance in the cases which leads us to infer that unobserved events have happened, are happening, or will happen." Sometimes one case is enough to produce the inference; at other times a collection and comparison of various instances is necessary, before we can conclude. A good example follows:—"From what may be conveniently termed the collective fact, that men have hitherto been fallible as far as observation has extended, I may deduce the particular conclusion, that an unknown and untried individual named Peter is fallible, and I may equally deduce the universal conclusion that all men are fallible."

There can be no doubt, we apprehend, of the general soundness of the above remarks: they present, in fact, examples of induction—not indeed of Aristotle's induction (ἐπαγωγή) or syllogism by induction, in which there is a professed enumeration of all the particulars; for he concludes, oddly enough, that "the whole class of animals wanting bile are long-lived;" but this only by enumerating, as he supposed, all the species of animals of that class. It is obvious that it is in this way alone, theoretically, that induction can be absolute, and have precisely the same kind of force which is due to the ordinary deductive syllogism. In the latter case we deduce the new particular from the general, which is asserted to include all the particulars; in the case of perfect induction, we build up the general out of all the particulars which are included under it. But we can only do this in strictness when we really know all the particulars; which we very seldom do. Our induction therefore is, for the most part, imperfect—that is, it is not apodictical or demonstratively absolute. Hence the inductions of science only amount to probabilities, however high. In Mr. Bailey's language, they are contingent. Thus, though all naturalists believe that "all horned animals have cloven feet," because no living or fossil animal has been found to exhibit a different law; still, it would not be absurd to suppose that an animal *might* possibly be found without a combination of these conditions. In the same manner we believe that every individual now alive will die; but, so far as this conclusion is matter of human reason, we believe it, not on demonstrative principles, but on the ground of probable ("contingent") evidence, amounting, no doubt, practically, to moral certainty. It should, however, be remembered that although the general conclusion which we build up from particular instances cannot have the force of positive demonstration, unless all the particulars are enumerated; nevertheless, when once we have admitted the general principle as a major premiss, and have referred any class of objects to this premiss as a minor, we are entitled to pronounce the universal law or attribute predicated in the major premiss, to belong as matter of necessity to any given particular included under the minor premiss. If you assert that all men are mortal beings, and if you further assert that a certain class of beings are partakers of human nature, you are compelled by the laws of thought to assert that any individual, however unknown to you, who belongs to that class, is of necessity mortal. If there be in the conclusion any want of absolute certainty, such as mathematical demonstration involves, this defect lies, not in the connexion between the premises and the conclusion, but in the principle or universal major premiss which you have admitted into the reasoning; or, in other cases, the other premiss may be at fault.

We must hear what our author says on this subject. He denominates the proposition, "*all men, as far as observation has extended, have been found fallible,*" "the collective fact." The "universal law" inferred is, "therefore all men are fallible;" and the assertion, "*therefore the man Peter is fallible,*" is termed "the particular inference." He proceeds :

"Both these conclusions are deduced from the same fact or collection of facts: they are co-ordinate; one is not or needs not be logically subsequent to the other; both are probably inferences, for which the real evidence is the same. The mental process, too, is alike; it does not consist in the mind's discerning one thing to be implied in another; but in its being determined by known facts to believe unknown facts."

If by this be meant, that a certain collection of facts frequently causes the mind to form a general principle which includes all particular cases, we can have no hesitation in admitting it. But if this language mean, as it seems to do to us, that the psychological process by which we obtain the general principle, is precisely similar to that by which we assert a particular case, we should demur to it. No doubt, logically, the particular is contained in the general; but the question is—how does this appear? What is the process of which we are conscious? The examples of horned animals having cloven feet are very numerous. They have occurred under a great variety of circumstances. We assert that all horned animals have cloven feet. We are prepared to expect that whenever we see an animal with horns, it will also have cloven feet. But we may be unacquainted with many kinds of animals of this class. We do not profess to have included all in our actual examination; if we did, our induction would be a perfect one; it would be demonstrative, and not contingent. As it is, we have, after obtaining a number of instances, advanced *per saltum* to a universal proposition; and we are now, and not till now, in a condition to say that all future instances may at once be disposed of by being brought under this proposition. It is very true, that in an example of perfect induction we do obtain an identical proposition, corresponding in its place to the minor of a deductive syllogism; for instead of the form all As are Bs, all Cs are As, therefore all Cs are Bs; we have the following: x, y, z are D, x, y, z are (whole) E; therefore E is D; but we do not see that any right exists to pronounce on any particular example (where we have not enumerated all) until we have become convinced of the general principle; for, until we feel ourselves justified, by some means, in waiving all further induction, and neglecting what remains of the series of examples, we do not feel assured that the very next instance may not stop us in our career, and prove that our embryo principle is wrong. Notable instances of this kind have actually occurred, even in the more exact branches of science, where general proofs were either not discovered or unattainable; of which an example occurs in Euler's "Memoirs of Berlin." We would suggest that a distinction between the logical and the psychological would often help us where there may appear a conflict of seeming truths. Thus the *infinite* logically contains the *finite*, but can it be doubted that psychologically and in point of fact we acquire a knowledge first of the finite, and by this are led on to form some notion of the infinite? So, also, it is certain that a universal proposition logically contains all the particulars which may turn out to belong to it; but this does not prove that we do not arrive at particulars never before known, through the previous admission of the universal, gained by ordinary induction—indeed, here, we need not restrict the theory to induction, for the principle is equally true of the demonstration of particular cases when they are brought under general and *à priori* truths. That "every act of reasoning proceeds on some general principle" must be granted: but when our author states, that the reasoning by which we conclude that "all the persons walking on the beach must sooner or later die," may be thus expressed: "All human beings formerly living have died before attaining a certain age; therefore these human beings will die before attaining that age"—the conclusion, though it is no doubt connected with the understanding that like effects proceed from like causes, is, we would suggest, only so connected through the medium of the general principle. We believe that A, B, C, now "walking on the beach," will die, not merely because an innumerable multitude of human beings have died; but because we cannot but suppose that those who have died sufficiently represent all mankind, among whom all future individuals must be classed. Our particular conclusions all virtually pass through the general principle.

Our author next inquires, how the cogency of this "contingent" reasoning—for "it is confessedly not demonstrative"—is to be proved. We agree with him that there can here be no demonstrative proof. If there could, the reasoning would no longer be based on general induction; it would either exhibit one of the few cases of perfect induction, or it would be at once deductive in the strictly demonstrative sense. When we say this, we do not forget what we have before said, or at least fully implied—namely, that when once we have assumed that our general principle may be taken as though absolutely universal, there is no distinction in the mode of inference. If any one asserts that all magnets attract iron-filings, he is as much obliged, by the laws of thought, to admit that this, that, or the other magnet will attract iron-filings, as he is obliged to admit that any possible plane-triangle that can be drawn will have its angles together equal to two right angles, after having once admitted that this is true of the general scheme or form *triangle.* But it must still be granted, that whatever absence of apodictical certainty may attach to the general principle, must attach to every particular case under it. Our author is, we think, quite right in referring to some principle of our nature, of a character which might be almost called "instinctive," the tendency we have to believe that what has happened will, in like circumstances, happen again. It is a fact that animals act on this principle. The dog which has repeatedly been thrown into the water, may be observed to give evident indications of apprehension on approaching a river. We confess we should be inclined to make a distinction between this case—as related to the operation of the tacit principle, "like causes produce like effects"—and the case of children, after reason and reflection have been developed. Animals, we apprehend, are influenced in these instances by association; but in man, this principle being blended with reflection, the result is not a blind impulse, but an inference guided and modified by intelligence. I have experienced many times the fact, that a stone thrown up into the air falls to the ground; and I learn that the same fact has been observed from time immemorial. Many more stones will be thrown up—what will be the result? always they will fall. Hence the general law will operate in any particular instance you can name. Had association alone been adequate to solve the psychological fact of my thus concluding, there would have been no place for a general principle; but reflection and inquiry show so many uniform cases, that the uniformity is predicted to be continuous, and we may therefore bring any particular case under it. No rule can be given, we must admit, for the point at which the collection of facts shall stop, and be pronounced adequate as a basis for the general law. Different experimental sciences demand different precautions and tests; but there is always a point beyond which we should feel all further experiment or inquiry to be superfluous; and, at this point, the principle is seized and held with a tenacity which we are very well content to term the result of a law of our mental constitution—call it a sort of intellectual "instinct"—call it a species of intuition, if you please. We are also well satisfied with Mr. Bailey's summation of his doctrine, in the brief form, that "when our minds are determined by present facts, conjoined with experience or knowledge, to believe some fact past, absent or future, we reason;" but we would repeat, that we distinguish this reasoning from mere *unreasoning* association. The child who has been once burnt "dreads the fire;" the man who knows by long experience the properties of fire, is certain that he will be burnt if he thrusts his fingers among live coals, because he has learned that fire always severely punishes those who trifle with it—he believes in a general law.

Mr. Bailey's third chapter treats of Demonstrative Reasoning. The instance given is, in fact, Euclid's axiom respecting the equality of things respectively equal to the same thing; or, at least, it is an example of this axiom.

"The mind, observing successively the equality of A to C, and that of B to C, is thence led to discern the mutual equality of A to B, which is not self-evident, or immediately discernible from the inspection of A and B alone. It is plain that in reasoning of this second species, which is with great propriety termed demonstrative, we intuitively discern, at each step, that one fact implies another, and discern, too, that a denial of the implied fact involves a contradiction."

Now here, we should say that either too little is stated, or too much. If the author intends that Euclid's "axiom" is an example of demonstrative reasoning, we should deny this altogether. This would certainly be asserting too much. If it be meant that, in any particular previously unexamined case, such as that of some one A, B, and C, the assertion is true—we ask why? The reply must be, surely, because it is impossible we should think otherwise. Is it again asked—*Why so?* what other reply can be given than that it must be so in *all cases,* of which this is one. In fact, we hold the axiom, as a general principle, to be like all other *real* axioms, whether geometrical or other, to be not only self-evident, but, at the same time, incapable of proof. Now surely no one has ever demonstrated the axiom that things equal to one and the same thing are equal to each other. Every mind which once comprehends the terms in which this proposition is couched, instantly feels it to be true, and, on a little reflection, also feels that no proof of it is possible. It is a truth which is on a par with many others, which we believe only just because we cannot help it. Such a truth is that of our own personal consciousness and existence; or that of the necessity of causation for every change. But when we say A is equal to C, and B is equal to C, therefore A and B are equal, we are doing nothing more nor less than giving an example of a principle on which, from early childhood, we tacitly act every day. This is evident when we consider that if any one should question our conclusion, we instantly justify it by saying, Why, must not things respectively equal to the same be equal to each other? We suggest, therefore, that our author has only given a partial statement, in the passage before us, of a case of demonstrative reasoning. His example is a case of demonstration *only,* because there is a tacit understanding of the general principle—the *à priori* truth that equals to equals are equal—a truth which would never have entered the mind but for some particular example, but which, on occasion of some such example, instantly flashed upon the understanding as universal and necessary. The reasoning, therefore, we hold to be as follows:—A and B are equal to each other if severally equal to C, *because* it is impossible that any case can arise in which equals to the same are unequal.

The above example is not exclusively geometrical, the only two distinctly geometrical axioms being, "two straight lines cannot enclose a space," and "only one parallel to a line can be drawn through a point outside it;" and we agree with Mr. Bailey, that "demonstrative reasoning is not confined to the science of quantity." By demonstrative reasoning, we imagine, from the context, that he would mean that which is, throughout, absolute and necessary in its conclusion, not only from the conclusion being drawn, strictly, from the admitted premises, but from the premises themselves being also incapable of being doubted. One example, however, is given, which does not fulfil this condition, and which ought rather, on Mr. Bailey's own principles, to have been regarded as a case of "contingent reasoning." The example we refer to is: "All horned animals are ruminant;" therefore "this horned animal is ruminant." No doubt the conclusion, here, follows necessarily from the premises; but so also does the conclusion, "The man Peter is fallible," follow necessarily from the expressed premises that "all men are fallible," and the implied premises expressed with the conclusion, that Peter is a human being. Peter's fallibility as necessarily follows from that of all mankind, as the rumination of any one horned animal that has, does, or shall exist, follows from the assertion that all horned animals ruminate. The difference, therefore, between the two cases would naturally be sought by the reader in the character of the major proposition. But it is evident that in both cases this proposition is not an *à priori* truth; it is, as Mr. Bailey calls it in the former case, a "collective fact," and it is equally so in the latter. We should hardly, therefore, have expected that the general truth, "all horned animals are ruminant," would have been classed with the general truth, "the three angles of every triangle are together equal to two right angles." The one is no doubt a contingent fact, that is, a fact of induction; the other is demonstrable *à priori*.

We are glad to find, notwithstanding certain modes of statement in this work, in which, as our readers have already seen, we are not entirely at one with Mr. Bailey, that he vindicates the syllogism in demonstrative reasoning from some of the charges brought against it by some writers of note, who appear to have mistaken its pretensions, and to have taken a wrong view of it in relation to the psychological process which it exhibits. We quote the following passage:—

"The objection is that the major premiss not merely implies but contains the conclusion; that the conclusion is in reality a constituent or integrant part of the major premiss, without which the latter would not be completely true. This allegation, it must be confessed, cannot be contradicted. The force of the reasoning in a demonstrative syllogism, or an enthymeme with a major premiss, depends altogether on the fact expressed in the conclusion, forming an integrant part of the general fact expressed in the major proposition, and consequently no new or unknown fact can ever appear as the inference. The essence of the conclusion, in such cases, consists in asserting that the subject of it *does* form an integrant part of the major premiss. But although the allegation must be admitted, it does not by any means prove that such reasoning is nugatory or useless. It may obviously be of service to be reminded, or to remind others, or to have distinctly brought into view, that a given individual of a class possesses a certain attribute, when there is at the moment no other evidence to prove it, by citing the known or admitted fact, that all the members of the class possess it. As an illustration of this part, suppose I am engaged in the demonstration of a geometrical theorem: there is before me a complicated diagram containing, amongst several figures, a triangle, which I have to compare with other figures, and, as a step in the reasoning, I have to show that the angles of the triangle in question are together equal to two right angles. I have not gone through the proofs with this particular triangle, but I call to mind that I have seen the proposition demonstrated of all triangles whatsoever; and from it, as an established truth, the conclusion that the angles of the triangle in the diagram, though not expressly investigated, are together equal to two right angles, irresistibly follows. It is simply thinking or saying: 'in all triangles, the three angles are equal to two right angles, and of course, the particular triangle before us is included in the general fact.'"—pp. 39, 40.

The above we hold to be good orthodox doctrine; but we must demur to a subsequent statement, that "all instances of the conversion of propositions are really instances of demonstrative reasoning." We should rather say, that if all As are Bs, it intuitively follows that some Bs must be As, without the intervention of any additional proposition expressed or implied. When we say "all men are mortal beings," we say that there are beings which have the two marks, *human nature* and *mortality;* but we do not say that wherever either of the marks exists, the other is also found. We simply shut up men within the sphere of mortal beings: we do not say, in stating this proposition, whether the sphere of men is coincident with that of mortal beings or not. On the contrary, when we say "no man is an infallible being," we are entitled to say "no infallible being is a man," simply or sufficiently because the terms mutually exclude each other by the very form of the expression—we deny all intercommunion between them. When we have said no As are Bs, we have said, in fact, that no Bs are As, and *vice versâ.*

We should also equally except, again, to Mr. Bailey's mode of getting rid of premises, in some cases, where he alleges that their introduction "masks the real nature of the evidence for the conclusion." He objects to the syllogism, *all horned quadrupeds are ruminant, and therefore this animal, being horned, will also be found ruminant,* his objection being founded on the above reason; and he states that the real argument is—"All *other* horned quadrupeds have been found ruminant, therefore this horned quadruped is ruminant." Now we think the major premiss, here, is hardly stated fairly. Taken literally, it means, first, that we have had actual experience of all horned animals *but one,* and have found them ruminant; and therefore, having now found the last horned animal, we conclude that it also is ruminant. This induction would be almost a perfect one, the collective fact having a force approximating by so much nearer to absolute certainty as the known cases are more numerous, or have fewer unexamined cases. But *is* this the real major premiss? or does it express the real collective fact? We think not. The real fact is, not that all other horned animals (this one excepted, as being yet unknown,) have been found—and have been found *ruminant;* but that all which have hitherto been found have been ruminant. Hence the mind, by some process natural to it, but not capable of much analysis, flies to the general principle, that horned animals always will be found to ruminate; that this, in short, is a law in natural history, and hence the conclusion, be the particular case what it will; and although it be admitted that not a millionth part of all the horned animals in creation have been, or ever will be, examined by man.

We have already admitted that the conclusion, in the case of ordinary inductive or "contingent" reasoning, must partake of precisely the same degree of probability as the "collective fact" laid down as the major premiss, though this conclusion as *necessarily* follows from the premises as any conclusion deduced from an absolutely universal major premiss. Whether this reasoning is to be called "demonstrative" or not, is a question of terminology. The brief dialogue which is introduced in the fifty-second page by way of illustration, would evidently be as applicable to any reasoning that we could call demonstrative, as to the contingent example there adduced; and we might parallel that example by one taken from the chapter on "Demonstrative Reasoning," and say, the three angles of every triangle, including the triangle A, B, C, are together equal to two right angles; therefore the triangle A, B, C, is equal to two right angles. But this would not, we imagine, be a fair representation of the true psychological analysis of the syllogism. We want to develop a process, step by step, which is for the most part hurried over practically, so as seemingly to contain merely two propositions, to the latter of which the mind is necessarily determined by the former. But a little reflection will show, that when we have got a major premiss, it may be still often necessary, for the sake of clearness, to state a minor, or, in other words, to announce that a certain individual or class actually does belong to the class of which something is predicated in the major premiss.

We do not think that our author has quite done justice to the subject of *axioms,* though he has fortified himself with the names of Locke and D'Alembert. Locke, in terms, denied innate truths or principles, though he admitted them in practice, and was in so doing notoriously inconsistent with himself; as every tyro knows who has carefully read his Essay. If it be true to say, with D'Alembert, that there is "no necessity to enunciate axioms," certain it is that we cannot get on a step without proceeding on the principle that there are axioms. They are always tacitly presupposed, and if we are to have anything like a full analysis put down on paper of the process of the mind in reasoning, it is necessary, in some cases especially, to state axioms at length. Mr. Bailey says that maxims or axioms are "only generalizations of the particular arguments, or of the particular instances of implication, and the self-evidence of both maxims and arguments is on a level, although the priority in respect of origin is with the latter." There may be some ambiguity, perhaps, in the meaning of this language; but if it mean that it is only after a certain number of arguments have been felt to be conclusive that we form out of them a maxim or axiom, we should say that this is only true of ordinary induction, in which the term axiom would not be rightly used; for an axiom, as already remarked, is properly a proposition which is both self-evident and incapable of proof. It carries its own conviction with it, and it admits of no corroboration. Let us take the former axiom, "things equal to the same thing are equal to each other," which is adduced by our author as an example of his view that axioms are only "generalizations of particular arguments." If by "priority of origin" it is intended to say that some example of the comparison of equal things with a third must arise, before the mind frames the axiom to itself in distinct consciousness, we admit it. But is it not true that the very first time that an intelligent child has such an example distinctly brought to his attention, he would admit the truth just because he feels, or, if you please, discerns at once by the eye of reason, that it cannot be otherwise—that is, that if A and B are each equal to C, they must equal each other—let A, B, and C represent what they may—that is, no case is conceivable, whatever be the equal things, or the nature of the equality, in which they must not both be equal or both unequal to the same third thing. If this be a mere "generalization of the particular arguments, or of the particular instances of implication," we would take the liberty of asking one question, and when it is answered we shall know whether or not we ought to give up our opinion of the nature of real *axioms*. The question is this: How comes it to pass, that, quite "irrespectively of the mere number of the instances, the generalization of the particular arguments" is, in some cases, felt to lead only to probability, however high, while no absurdity seems to attach to the idea of an instance occurring in which the principle shall fail—whereas, in other cases, it is at once felt to be absurd to imagine the possibility that any instance should ever occur in which the principle is not true. It involves no absurdity to imagine that a horned animal were found without hoofs, but what should we say of any one who gravely maintained that a change might take place without a cause, or that an instance was possible of two things, each equal to a third thing, in the same respect, while the two things are, in that respect, unequal to each other. The fact is (or at least so it appears to us) that in the former case the general principle has grown out of a great number of instances, each of which has added to its probable truth; while in the latter case the general principle was felt to fasten on the mind (on the first particular example occurring) with all the force of universality and necessity. In one sense it is true that "maxims (axioms) have no probative force, they add no cogency to any argument;" for the argument, in fact, already presupposes their tacit admission: but if they do not add force, they point out where the force lies. The ground we tread on adds no vigour to our muscles in walking, not previously inherent in them; but it renders walking possible; in other words, (though it is so obvious as to be readily overlooked,) the very idea of walking presupposes or involves that there is always something to walk on. Archimedes not only wanted machinery, but also the πoῦ στῶ.

That Aristotle's *dictum* is a "self-evident and indisputable truth," we readily admit; and we would go further, and say, that it may be reduced to the assertion—call it definition, or axiom, or what not—that every whole includes all its parts. All we have to do is to determine, and if needful to assert, that a certain individual *is* a part of the given whole, and the conclusion. We may laugh at the simplicity of this doctrine, if we please, and say that no ghost was needed to tell us that. Well and good: but the only question is, whether this is not the true psychological analysis of reasoning. No doubt, as Mr. Bailey says, "When I affirm that a man could not commit a crime at a specified time in London, because he was at that precise moment in Edinburgh, I reason just as much as I do when I affirm—that the three angles of the triangle before me are together equal to two right angles, because the three angles of every triangle are equal to two right angles." Yes, certainly, I reason as much in the former as in the latter case: but what is the principle of my reasoning—or has it any principle? Imagine a person to be asked why a crime could not be committed in London under the above circumstances, and what is tacitly proceeded on, as the ground of conviction in the particular instance, would at once be stated in words—namely, that it is impossible for any person to be in two places at the same moment. Would not this show the granted or understood general principle on which each particular case was based?

No logician will be prepared to deny that maxims or principles may be drawn out briefly expressing the precise character of the syllogisms in the imperfect moods, as has been done in the *Organon* of Lambert, a German logician of merit. Mr. Bailey has done the same; but the question is not so much, how we may describe certain arguments, but whether there be any way in which all may be described. It would be impossible to reduce all kinds of categorical arguments to any of the four maxims proposed by our author; but by certain legitimate and obvious transpositions of premises, and conversions of propositions, in the process of which not an iota is uttered, or denied, which was not uttered or denied in the original state of the arguments, all are reduced to the simple form of the dictum; and many are, by this means, made much clearer, and more intelligible to the ear and mind.

Let us take the following argument in the fourth figure; which figure, by the way, is not Aristotelian, being traditionally ascribed to Galen:—All the planets are opaque bodies: no opaque bodies are capable of transmitting light in any other way than by reflexion; therefore bodies capable of transmitting light in any other way than by reflexion are not planets. Now, it cannot be doubted that the immediate principle on which this syllogism is constructed may be expressed, as in the *Artis Logicæ Rudimenta* (*Oxon*), thus: "If one class be universally comprehended under another, from which a third is wholly excluded, this third is wholly excluded from the first." But who would dispute the superior clearness of the argument in the following form, in which the same things are laid conformably with the *dictum?* No opaque bodies are capable of transmitting light in any other way than by reflexion. All the planets are opaque bodies; therefore the planets are not bodies capable of transmitting light in any other way than by reflexion —a conclusion logically identical with the former, as the terms mutually exclude each other. The dictum is truly not the sole principle which may be applied to legitimate arguments; but the dictum expressed in some way or other, so as to point to classes or attributes, has never, so far as we know, been fairly disproved to be a principle to which all syllogistic reasoning may be reduced, when put in its most analytical and elementary form.

We regret that our space forbids us to pursue further the analysis of the volume before us in detail. This, however, is of much less consequence than it might be, if the views which we have already examined did not frequently reappear, in the subsequent pages, by way of their further application. Our remaining observations must be concise, and almost aphoristic. Mr. Bailey remarks that, in this argument, "the planets are opaque bodies; therefore they must shine by light derived from an external source;"—it is obvious that a "proposition affirming that all opaque bodies shine by light derived from external sources, would be merely generalizing an argument sufficiently conclusive, and would not add to it a particle of cogency." Now, we must say that we deem this observation quite beside the mark. There has probably been many a student who, on first reading Newton's *Principia*, (if he studied the original text,) would have been glad if its illustrious author had been a little more detailed in his analysis; but no one who has read Euclid would suppose that even the minutest detail of steps would have added a particle of *cogency* to Newton's conclusions. The addition of a major premiss to the above truncated syllogism (which the moderns call an enthymeme, though it differs widely from the enthymeme of Aristotle) would certainly add no cogency to the argument, but is as certainly implied in the argument as such; for it is evident, that when we merely say that planets, as being opaque bodies, must shine by a light not their own, we imply that the planets are no exception to the general law of opaque bodies. We do not here stay to quarrel with the matter of this particular example; but we believe that the best dictionaries, and scientific usage, would warrant the application of the term "opaque" (not diaphonous) to the sun itself. The example we have just given, in the fourth figure, is evidently different; for an opaque body cannot transmit light *through* it.

In regard to our author's example: "Solon was a wise legislator, because he adapted his laws to the genius of the people"—what earthly connexion, we would ask, can there be between these two assertions, which does not imply that the conduct ascribed to Solon was a *mark of wisdom*, and would have been such *in any legislator?* Here then, surely, is an implied *principle;* which you may express if you please, but which you *must* tacitly admit, if your conclusion is to have any real connexion with the reason given for it. Our author, however, purposes, by way of "strengthening the reasoning," to add to it such a proposition as the following: "For when laws are adapted to the genius of the people, they are cheerfully obeyed." Now, this addition may be very good; but then it is evident that it will not answer the purpose wanted: for we shall now have two arguments instead of one; the first proving that Solon was wise because he adapted his laws to the genius of the people—the second proving wherein this wisdom consisted—namely, in taking the readiest road to have the laws obeyed. The question is simply whether it is worth while or not to have an exact psychological analysis of a process of argument, understanding by an "argument" a case in which some one proposition is made to follow from what preceded? It would be pedantic enough, no doubt, and very tedious, to supply all the enthymemes in which we talk, with the omitted premises; but this proves nothing against the *dictum* as a register of the actual or virtual psychological process. It would be just as bad to parse every word in our conversation; nevertheless grammatical analysis is not without its use, and philology is an important science.

Our author concludes his discussion of the "forms of reasoning" with the remark that—"in numerous cases of demonstrative reasoning, one premiss is alone sufficient for the inference; although it may be granted that, even in those cases, it is possible to form a complete syllogism, by thrusting in a fruitless and redundant proposition." We grant that there are cases in which it may be much more important, for the sake of clearness, to state an omitted premiss than in others: we go further, and admit that many logical examples appear frivolous, and almost ridiculous, when stated at full length, chiefly because the thing to be proved and the premises which may be stated in connexion with it happen to be *so very familiar*. Every body knows that "all men are mortal;" and every body who knows "Peter" knows that he "is a man," and not an angel; and every body knows that "Peter is mortal:" but will any body deny that the statement of Peter's mortality is true, *because* Peter is a member of the human family, and so comes under the general law to which, from the beginning, it has been observed that mankind have been subject? Now the syllogism merely says this in all the minutiæ of detail. The same remark, of course, applies to reasoning in which the major premiss is an absolutely certain or *à priori* truth.

To Mr. Bailey's conclusion, that "the syllogism is not an analysis of the process of all demonstrative reasoning;" and "that a single fact or combination of facts capable of being expressed in one proposition, frequently determines the mind to a conclusion without reference to anything else;" and that this is the whole of which the mind is "conscious, or which can be discerned as having taken place on reflection"—to this, we need not add, we decidedly demur. It may include a description, perhaps, of what takes place in cases of mere association, which often misleads—witness Bacon's four "idols;" but it is surely a very inadequate, we may say incorrect, account of what takes place in reasoning proper, and more especially in that which is demonstrative. In fact, it wholly overlooks the sense, tacit or expressed, of a *general law.*

We would willingly have pursued our analysis of the author's volume throughout, because his book is really a good one of the school to which it belongs; but we must forbear. In the chapter on "Primary or Original Premises," he fortifies himself with references to Locke, Dugald Stewart, Smart, and John Mill. With the latter work he shows that he is familiar, and to its views some of his own will be thought by the reader to bear considerable resemblance. We were glad to find him, in his chapter on the "Relation between Reasoning and Language," controverting Whately's statement, that "logic is solely conversant about language"—a statement not in harmony certainly with Aristotle's doctrine of the syllogism; and, what is much more important, not in harmony with consciousness, as Hobbes, Brown, and perhaps Dugald Stewart, in some measure, were aware. In the tenth chapter are some valuable remarks on the "Relation of Observation, Experiment, and Induction, to Reasoning and to each Other." The eleventh chapter is practical, containing "Rules for Guiding the Operation of Reasoning." It was to be expected that here our author would hardly be prepared to do justice to the scholastic logicians, who, with all their faults, were not without great merit—witness the "Manuductio ad Logicam" of Du Trieu. The volume closes with some useful popular remarks on the "sources of erroneous conclusions;" but the author does not go into a minute exposition of sophisms. An Appendix follows, containing some analyses of trains of reasoning; in which, after all the criticism which the *dictum de omni et nullo,* and the syllogism have undergone—enough surely to lay these ghosts for ninety-nine years—they nevertheless reappear, and are allowed to possess a vehicle of corporeity; for they are both referred to as substantial elements of reasoning, and especially the former, though it is evident that they must stand or fall together.

We will only add, that we apprehend much of the controversy which has taken place on the fundamental principles of logic has been occasioned by the contending parties not fully comprehending each others drift and aim. Moreover, the psychological aspect of a process of consciousness, and its logical content, though they can never really clash, not unfrequently present apparent discrepancies, and require to be harmonized, as they often easily may. The distinction has been well marked in the modern Eclectic school of France. The use of terms, again, is constant *crux* of logicians, as well as of divines, moralists, and metaphysicians. These circumstances have all tended to augment the difference subsisting between those who are more inclined to Aristotelian views of logic, and those who would like to banish the Stagyrite wholly from his throne.

